# Disappearance of *Anopheles minimus* and *Anopheles dirus* from Certain Malaria Endemic Areas of Assam, India

**Published:** 2017-03-14

**Authors:** Kavita Yadav, Sunil Dhiman, Bipul Rabha, Diganta Goswami, PK Saikia, Vijay Veer

**Affiliations:** 1 Medical Entomology, Defence Research Laboratory, Tezpur, Assam, India; 2 Zoology Department, Gauhati University, Guwahati, Assam, India

**Keywords:** Mosquito vectors, Malaria, Japanese encephalitis, Ecology, India

## Abstract

**Background:**

Orang Primary Health Centre (OPHC) and Balipara Primary Health Centre (BPHC) of Assam (India) report mosquito borne diseases annually. Current study was performed to ascertain the prevalence of known malaria and Japanese Encephalitis (JE) vectors and their possible role in disease transmission.

**Methods:**

Malaria epidemiological data for 2006–2010 and JE data for 2008–2013 of Assam, India were obtained from the health authority. Mosquitoes were collected using CDC light traps and identified morpho-taxonomically.

**Results:**

*Plasmodium falciparum* cases (81.5%, 95% CI= 72.0–91.1) were statistically higher in OPHC (P< 0.0001, t= 8.0) during the recent years. There was 4.4 folds rise in the confirmed acute encephalitis syndrome (AES) and 3.2 folds increase in the confirmed JE cases during 2013 as compared to 2008. Altogether 9,218 mosquito specimens (PTND= 153.6), comprising of 44.1% anophelines (PTND= 67.7), 42.3% culicines (PTND= 65.0) and 9.5% mansonia (PTND= 14.6) were recorded. In BPHC, *Anopheles vagus* was recorded in high density (P< 0.0001), whereas *Culex quinquefasciatus* was the predominant JE vector (P= 0.04). In OPHC, among the known malaria vectors, the density of *Anopheles annularis* was significantly high (P< 0.0001). However *Culex bitaeniorhynchus* was the predominant known JE vector (P< 0.0001) followed by *Cx. quinquefasciatus.*

**Conclusion:**

Even in the absence of known efficient vectors, many *Anopheles* species are still involved in malaria transmission. There was disappearance of *Anopheles minimus* and *Anopheles dirus* and establishment of *An. annularis*, *An. vagus* and *An. philippinensis/nivipes* mosquitoes in study area.

## Introduction

Mosquitoes spread pathogenic agents of malaria, Japanese Encephalitis (JE), dengue, lymphatic filariasis and chikungunya in many countries. The data on prevalence of known mosquito vectors constitutes vital and useful information to control the mosquito-borne diseases. Despite concerted vector borne diseases intervention efforts in India during 2014, approximately 1.07 million confirmed malaria cases and 535 deaths have been reported, while 1,661 confirmed cases and 293 deaths have been attributed to JE (NVBDCP 2014).

Mosquitoes are remarkably adaptable insect group, which continue to successfully coexist with human being and survive by feeding on human host and his domestic animals in addition to plant nectar. Assam is the largest state (population wise) in northeast region of India, where malaria transmission is endemic. In India, *An. minimus* Theobald, 1901, *An. dirus* Peyton and Harrison, 1979, *An. fluviatilis* James, 1902 and *An. culicifacies* Giles, 1901 have been recognised as potential malaria vectors, while *An. annularis* Van der Wulp, 1884, *An. philippinensis* Ludlow, 1902 and *An. varuna* Iyengar, 1924 play limited role in malaria transmission (Dev et al. 2003, Prakash et al. 2004, Bhattacharyya et al. 2010, Dhiman et al. 2011, 2012). JE outbreaks are common in northeastern states including Assam and occur mainly during rainy season. Sixteen mosquito species have been incriminated as JE vector in India, of which *Culex tritaeniorhynchus* Giles, 1901 and *Culex vishnui* Theobald, 1901 subgroup has been reported as prominent JE vectors in the endemic areas (Kanojia 2007, Saxena et al. 2008, Dhiman et al. 2013). Many culicine and mansonia species, namely, *Cx. vishnui*, *Cx. bitaeniorhynchus* Giles, 1901, *Cx. gelidus* Theobald, 1901, *Ma. uniformis* Theobald, 1901, *Ma. annulifera* Theobald, 1901 and *Ma. indiana* Edwards, 1930 are well known vectors of JE and reported in many parts of Assam and other north-eastern states of India (Saxena and Dhole 2008, Dhiman et al. 2009, 2013).

Udalguri and Sonitpur districts of Assam are highly endemic for malaria and contribute considerably to the malaria cases in the state. A recent study conducted in Udalguri district has indicated that, the number of health centres having annual parasitic index (API) of > 5 and more than 30% of malaria cases due to *P. falciparum* Welch, 1897 were increased in the recent years. The study exhibited that 11 health centres were malaria hot spots, of which 9 were part of Orang Primary Health Centre (OPHC) (Yadav et al. 2012). Similarly, in Sonitpur District 10 health centres including Balipara have been identified as malaria hot spots, which have an extremely high malaria risk (Nath et al. 2013). The forestlands of both the districts have remained the areas of intense malaria parasite reservoir providing foci for re-infection in the other neighbouring areas. Moreover, ecological changes due to deforestation have brought some changes in geo-climate that has significantly influenced the mosquito vector ecology and diseases transmission (Nath et al. 2012).

The dynamic distribution and transmission of malaria and JE in Assam poses a serious epidemiological challenge due to various socio-economic, geo-political and environmental factors (Dev et al. 2003, 2010, Dhiman et al. 2011, Rabha et al. 2012, Yadav et al. 2014). Information on vector entomology is an essential component in disease management, which depends upon the knowledge of vector species density and composition. Many studies have been conducted in various parts of the state, but anthropogenic ecosystem modifications in the past few years might have influenced the known malaria and JE vectors composition. Therefore, it is inevitable to update data on prevalence of vector mosquitoes for reviewing vector control strategies.

The present study was undertaken during April 2012 to August 2013 in malaria endemic Primary Health Centres of Udalguri and Sonitpur districts of Assam to generate information on known mosquito vector prevalence.

## Materials and Methods

### Study area

Current study was conducted in randomly selected four sentinel survey sites each (Table 1) in OPHC of Udalguri District and Balipara Primary Health Centre area (BPHC) of Sonitpur District. OPHC (92° 07′–92° 22′ E longitude and 26° 33′–26° 56′ N latitude) situated at 105.2 meters, is dominated by various ethnic tribes primarily engaged in agriculture and tea cultivation. The climate is sub-tropical humid and experience an average annual rainfall of about 2,000mm, while the temperature and relative humidity varies between 34.5 °C to 13.5 °C and 65 to 90% respectively. Study area has many small rivers, scattered tea gardens and vast paddy fields, which create suitable breeding ecology for mosquito vectors. BPHC (92° 38′–92° 59′ E longitudes and 26° 41′–27° 02′ N latitude, 74.7 meters) is dominated by different ethnic groups, including Bodo, Nepali, Aadivasi and Assamese, with agriculture based very low socio-economic status. The average temperature ranging from 15 °C to 35 °C, about 1,900mm average rainfall and 55–90% relative humidity plays a major role in determining the climate of the area. There are many rivers and large spreads of tea meadows and paddy fields. The prevailing climatic conditions are conducive for the breeding and proliferation of different vector mosquitoes. A recent study conducted in Sonitpur district using normalized difference vegetation index (NDVI) has shown that the forest-covered area was 1.2 folds decreased in two years (Nath et al. 2012).

**Table 1.** Global Positioning System coordinates of the sentinel survey locations (S1-S4) in both study areas of Orang Primary Health Centre (OPHC) and Balipara Primary Health Centre (BPHC) of Assam (India)

**Study area**	**Survey site**	**GPS location**
**Balipara Primary Health Centre (BPHC)**	S1	92°46'43.0″ E 26°41'18.6″ N
	S2	92°47'36.2″ E 26°42'02.3″ N
	S3	92°47'39.6″ E 26'40′35.3″ N
	S4	92°48'09.7″ E 26°41'31.8″ N

**Orang Primary Health Centre (OPHC)**	S1	92°16'15.8″E 26°38'23.8″ N
	S2	92°15'49.9″E 26°41'45.4″ N
	S3	92°17'32.4″E 26°41'52.0″ N
	S4	92°19'38.3″E 26°41'55.3″N

### Malaria and Japanese Encephalitis situation in the area

Malaria epidemiological data for the years 2006–2010 were collected from the District Malaria Office of the concerned district and analysed to understand the malaria situation during preceding years. Japanese Encephalitis data of Assam for the years 2008–2013 was obtained from the NVBDCP and depicted in Table 2.

**Table 2.** Past acute encephalitis syndrome and Japanese Encephalitis situation in Assam

**Year**	**Acute encephalitis syndrome (AES)**		**Japanese Encephalitis (JE)**	

	**Reported cases**	**Deaths (%)**	**Reported cases**	**Deaths (%)**
**2008**	319	99 (31.0)	157	33 (21.0)
**2009**	462	92 (19.9)	218	46 (21.1)
**2010**	469	117 (24.9)	142	40 (28.2)
**2011**	1319	250 (19.0)	489	113 (23.1)
**2012**	1343	229 (17.1)	463	100 (21.6)
**2013**	1388	272 (19.6)	495	134 (27.1)

### Collection and identification of known mosquito vectors

Mosquitoes were collected from dusk to dawn (1800–0600 h) with 6-volt battery operated miniature light traps (Centres for Disease Control, Atlanta, USA) and indoor resting using WHO aspirator tubes. The mud plastered kuchha houses and bamboo houses having a thatched roof and adjacent to the cattle sheds were selected for the study. The study area had numerous small ponds and irrigation channels, while number of domestic animals such as pig, fowl and duck were also available. Smoking and burning was prevented during the operation of the traps. The traps were installed near unscreened windows in the rooms at about 2 m above the ground and kept on throughout the night until removed in the early morning hour. The collected mosquitoes were etherised (if alive) and identified to species complex level with the help of standard keys (Barraud 1934, Wattal and Kalra 1961). Densities of known vector mosquitoes were calculated in terms of mean numbers of mosquito of a species caught per trap night and expressed as per trap night density (PTND) of that particular species.

### Data analysis

The collected mosquitoes were expressed in per trap night density (PTND), whereas known vector density for each species was presented as mean±SEM. Comparison of mosquitoes was performed using ANOVA followed by Tukey Krammer test of multiple comparison. PTND of known malaria and JE vectors was compared using unpaired students’ “t” test.

## Results

### Past malaria and Japanese Encephalitis situation

**Fig. 1.**
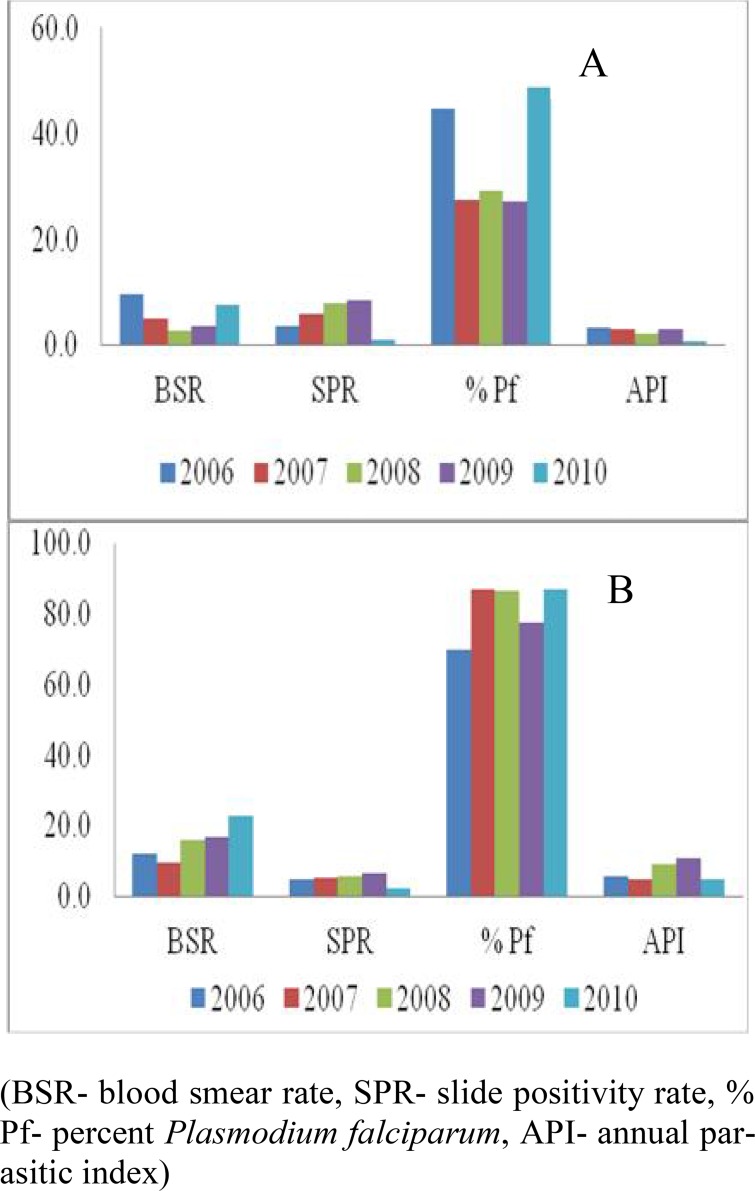
Past malaria situation depicting blood smear rate (BSR), slide positivity rate (SPR), % *Plasmodium falciparum* and annual parasitic index (API): (A) In BPHC, (B) In OPHC

Malaria situation during the past years (2006–2010) in BPHC and OPHC is shown in Fig. 1. The slide positivity rate (SPR) in both the locations was similar in the recent years (P= 0.8, t= 0.3), however cases attributed to *P. falciparum* malaria in OPHC were 81.5% (95% CI= 72.0–91.1) and found to be statistically higher than in BPHC (P< 0.0001, t= 8.0). Further, the average annual parasitic index (API) during the years 2006–2010 was 6.9± 12 (95% CI= 3.6–10.3) in OPHC as compared to 2.4±0.5 (5% CI= 1.1–3.7) in BPHC (P= 0.01, t= 3.4). Past encephalitis data suggests that as compared to 2008, there has been 4.4 folds increase in the confirmed acute encephalitis syndrome (AES) cases and 3.2 folds increase in the confirmed JE cases in 2013. However, confirmed deaths due to AES showed 2.7 folds while due to JE showed 4.1 increases during the last six years.

### Known malaria and Japanese Encephalitis vectors abundance

A total of 9,218 mosquito specimens were collected in the current study using CDC light traps and aspirators in 60 trap nights (PTND= 153.6). Of the total mosquitoes, anophelines were 44.1% (PTND= 67.7), culicines were 42.3% (PTND= 65.0), mansonia were 9.5% (PTND= 14.6) while remaining 4.1% (PTND= 6.3) corresponded to other mosquito species belonged to *Stegomyia*, *Neomelaniconion*, *Coquillettidia* and *Armigeres*.

In BPHC, total 7,201 (PTND= 180.0) mosquitoes constituting 37.9% anophelines (PTND= 68.2), 45.3% culicines (PTND= 81.6) and 12.0% mansonia (PTND= 21.7) were reported during the study. The density of anophelines and culicines mosquitoes was statistically higher than the other mosquitoes, however no significant difference was found among both of them (P= 0.8, t=0.3). Among the reported malaria vectors in India and neighbouring Bangladesh, present study found *An. annularis*, *An. culicifacies*, *An. philippinensis* and *An. vagus* Doenitz, 1902 mosquitoes. *Anopheles vagus* (58.1%, PTND= 37.7 of the total known malaria vectors) was recorded in significantly high density (F= 18.4, P< 0.0001), but did not differ statistically from *An. annularis* (P= 0.2, t= 1.6). Among the known JE vectors, *Cx. quinquefasciatus* Say, 1823 was predominant (F= 2.6, P= 0.04) and accounted for 49.5% of the total JE vectors (PTND= 44.6). Malaria and JE vectors density obtained in BPHC is depicted in Table 3. There was no statistically difference between malaria and JE vectors density (P≥ 0.3, t≤ 0.6) in BPHC throughout the study period.

**Table 3.** Known malaria and Japanese Encephalitis vectors in Balipara Primary Health Centre area

	**Species**	**Density (mean±SEM)**	**F (P)**
**Malaria vectors**	*An. vagus*	377.3±81.5	18.4 (< 0.0001)
	*An. annularis*	241.5±15.9	
	*An. culicifacies*	15.3±6.0	
	*An. philippinensis*	15.5±2.5	

**JE vectors**	*Cx. quinquefasciatus*	446.3±270.3	F= 2.6 (P= 0.04)
	*Cx. vishnui*	175.3±89.9	
	*Cx. bitaeniorhynchus*	120.8±100.2	
	*Cx. whitmorei*	25.5±4.2	
	*Cx. gelidus*	8.5±2.9	
	*Ma. uniformis*	131.8±115.7	
	*Ma. indiana*	30.3±19.2	
	*Ma. annulifera*	44.8±33.6	

In OPHC, of the total 2,017 (21.9%) mosquitoes in 20 trap nights (PTND= 100.9), 66.2 % were anophelines, 47.6% were culicines, while 1.8% corresponded to mansonia species. The PTND was highest for anophelines (66.8) followed by culicines (31.8). Density of anophelines was statistically higher than the other mosquitoes (F= 748.6, P< 0.0001). Among the known malaria vectors, *An. culicifacies*, *An. vagus*, *An. fluviatilis* and *An. annularis* were prevalent, however the density of *An. annularis* was significantly high (F= 180.3, P< 0.0001). *Culex bitaeniorhynchus* was the predominant known JE vector (F= 92.1, P< 0.0001) followed by *Cx. quinquefasciatus*. Malaria and JE vector density in OPHC is shown in Table 4. Malaria vector density was statistically higher than the JE vectors in OPHC (F= 15.1, P< 0.0001).

**Table 4.** Known malaria and Japanese Encephalitis vectors in Orang Primary Health Centre area

	**Species**	**Density (mean±SEM)**	**F (P)**
**Malaria vectors**	*An. vagus*	156.0±11.7	180.3 (< 0.0001)
	*An. annularis*	162.8±6.7	
	*An. culicifacies*	1.8±0.5	
	*An. fluviatilis*	3.5±0.6	

**JE vectors**	*Cx. quinquefasciatus*	63.8±7.7	92.0 (< 0.0001)
	*Cx. bitaeniorhynchus*	90.5±6.5	
	*Cx. whitmorei*	0.6±0.6	
	*Cx. gelidus*	3.3±0.8	
	*Ma. indiana*	0.8±0.4	
	*Ma. annulifera*	1.8±0.8	

**Fig. 2.**
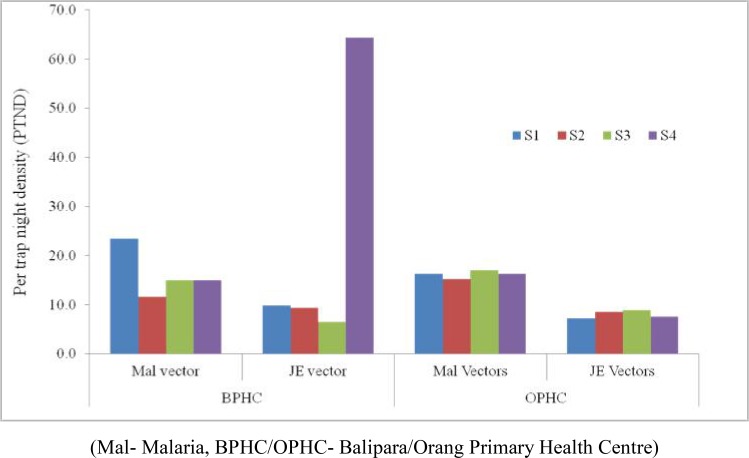
Per trap night density of known malaria and Japanese Encephalitis vectors in four sentinel collection sites (S1–S4) in BPHC and OPHC areas

Between BPHC and OPHC, the PTND of known JE and malaria vectors (Fig. 2) between both the study areas were similar (P≥ 0.4, t≤ 1.0).

## Discussion

Vector borne diseases control programmes have always stressed that up-to-date knowledge of spatial distribution and diversity of mosquito vectors across the endemic areas is inevitable for planning and implementing the effective intervention measures. In the current study, known malaria and JE vectors were collected in two ecologically distinct endemic primary health centres of Assam, where malaria and JE transmission is supported by many efficient mosquito vectors. *Anopheles dirus*, *An. fluviatili*s and *An. minimus* mosquito species have been considered as important malaria vectors, however in north-eastern states and neighbouring country Bangladesh the role of *An. annularis*, *An. culicifacies*, *An. philippinensis* and *An. vagus* in malaria transmission is determined (Prakash et al. 2004, Alam et al. 2010, Bhattacharyya et al. 2010, Dhiman et al. 2012).

The present study has indicated that many known malaria vectors were abundant in both the study areas, however could not report even a single specimen of established vectors *An. minimus* and *An. dirus* (Dev et al. 2003, Das et al. 2004, Sarma et al. 2012). Both *An. minimus* and *An. dirus* are known for uninterrupted spread of malaria in the region, but the surprising results of current study indicate that the other malaria vectors of comparatively lesser epidemiological importance might have taken over malaria transmission in absence of the well-established vectors. In BPHC of Assam *An. annularis* was found harbouring both *P. falciparum* and *P. vivax* Grassi and Feletti 1890 and its density outnumbered the density of *An. minimus* during malaria season (Dhiman et al. 2012). *Anopheles culicifacies*, although comes in lower counts but have strong anthropophilic character and malaria transmission potential in the suburbs of the region (Dhiman et al. 2012). Further, *An. nivipes* Theobald, 1903 and *An. vagus* have also been found positive for sporozoite in different malaria endemic areas of northeastern India (Prakash et al. 2004, Bhattacharyya et al. 2010). Presently, *An. annularis* and *An. vagus* were recorded in large number in areas where malaria is still endemic and many cases are reported annually (Rabha et al. 2012, Yadav et al. 2012, Nath et al. 2013). Although both these species are primarily considered zoophilic and exophilic in nature, but they have been considered to be opportunistic in the host selection for blood meal and have been thought to maintain malaria transmission in the region (Prakash et al. 2004, Dhiman et al. 2012). Large number of specimens corresponding to these two species was collected indoor resting which indicates that both of these might be shifting exophilic and exophagic behaviour to endophilic and endophagic. In Assam-Meghalaya border, *An. annularis* prefers resting indoors and a considerable proportion feed on human blood (Dhiman et al. 2014).

In the recent years, there has been tremendous deforestation, and new resettlements are coming up rapidly in the study area. The forestlands, which provide favourable breeding habitats for malaria vectors *An. dirus* and *An. minimus*, have been reduced significantly during last few years (Nath et al. 2012). Therefore, disruption in the ecology of these two vectors might have persuaded other anopheline species such as, *An. annularis*, *An. vagus* and *An. philippinensis/nivipes* to establish themselves as major species owing to the vast paddy cultivation in the area.

Among the JE vectors *Cx. bitaeniorhynchus*, *Cx. quinquefasciatus* and *Cx. vishnui* were recorded in large density, whereas *Cx. gelidus* was recorded in very low number. These vectors have been reported from other areas of Assam and found associated with the JE transmission in the region (Dhiman et al. 2013). In the past few years, the JE is emerging as serious vector borne disease in the entire north-east region, where reported cases and deaths during recent years has increased to many folds (NVBDCP 2013). However, the vector abundance of potential JE vectors has not been monitored regularly (Dhiman et al. 2013). JE outbreaks are common during the rainy season and occur at regular intervals in different parts of northeast region including study area. Therefore, data on population dynamics of JE vectors is important for focused control measures implementation. Mosquito density builds up during the epidemics, however abundance, survival and longevity of vector mosquitoes directly influence the dynamics of disease transmission annually.

The study area reports high incidence of malaria throughout the year. Various ethnic tribes having poor economic condition and their socio-cultural customs and beliefs make malaria vector control difficult (Dhiman et al. 2011, Rabha et al. 2012, Yadav et al. 2014). Malaria vector density was high in OPHC, which corroborates the high number of reported malaria cases. The API reported in OPHC is about 2.8 folds, while the percent *P. falciparum* is about 2.3 folds higher than in BPHC. Higher density of known vectors has been found associated with increase in disease incidence across many endemic settings (Alam et al. 2012, Dhiman et al. 2012). Although not much JE cases are reported in OPHC as compared to BPHC, but the tribal villages in the OPHC area have high pig density, which could serve as reservoir of JE virus, and also the high density of known JE vectors may sprout the disease during favourable transmission conditions. Ecological changes might have involved in the replacement of well-established mosquito species by other species. Deforestation and irrigation projects resulted in change of mosquito species involved in malaria transmission in Sri Lanka and Thailand (Amerasinghe et al. 1991, Prothero 1999).

## Conclusion

The present study is limited in its scope and reveals that variety of little known malaria and JE vectors are maintained in the study area, while some well-known vectors were disappeared or maintained at very low density. We have not attempted to incriminate any known malaria and JE vectors, but suggest that even in the presence of comprehensive vector control measures some little known vectors might have been playing a leading role in disease transmission. High density of known vectors may increase the risk of increasing insecticide resistance thereby circumventing the protection from insecticides. Further investigation on breeding ecology and insecticide susceptibility status of commonly used insecticides is important to provide information for adopting suitable control measures.
